# Whole-Genome Sequence of *Serratia liquefaciens* HUMV-21, a Cytotoxic, Quorum-Sensing, and Biofilm-Producing Clinical Isolate

**DOI:** 10.1128/genomeA.00533-15

**Published:** 2015-05-28

**Authors:** María Lázaro-Díez, Felix Acosta, Sara Remuzgo-Martínez, Alain Ocampo-Sosa, Javier Gonzalo Ocejo-Vinyals, Jimena Bravo, Fátima El Aamri, Oliver Escuela, Luis Martínez-Martínez, José Ramos-Vivas

**Affiliations:** a Servicio de Microbiología, Hospital Universitario Marqués de Valdecilla, Instituto IDIVAL, Santander, Cantabria, Spain; b Departamento de Patología Animal, Producción Animal y Tecnología de los Alimentos, Universidad de Las Palmas de Gran Canaria, Arucas, Gran Canaria, Spain; c Servicio de Inmunología, Hospital Universitario Marqués de Valdecilla, IDIVAL, Santander, Cantabria, Spain; d Red Española de Investigación en Patología Infecciosa (REIPI), Instituto de Salud Carlos III, Madrid, Spain; e Departamento de Biología Molecular, Universidad de Cantabria, Santander, Cantabria, Spain

## Abstract

A clinical isolate of *Serratia liquefaciens* (strain HUMV-21) was obtained from a skin ulcer of an adult patient. We report here its complete genome assembly using PacBio single-molecule real-time (SMRT) sequencing, which resulted in a single circular chromosome with 5.3 Mb. About 5,844 protein-coding genes are predicted from this assembly.

## GENOME ANNOUNCEMENT

*Serratia* spp. are opportunistic human pathogens responsible for an increasing number of nosocomial infections (1–3). However, little is known about the virulence factors produced by *Serratia* spp. that contribute to the pathogenesis of infections caused by these microorganisms, especially in *S. liquefaciens*. Previous studies using several *S. liquefaciens* clinical isolates have demonstrated the ability of this species to adhere to abiotic surfaces and to form biofilms that likely contribute to its persistence in the hospital environment (4). Moreover, in a previous report from our group, we demonstrated a fast and potent cytotoxic effect produced by a clinical isolate upon infection in macrophages (5). The strain used in this study (HUMV-21) was obtained from a skin ulcer of a man at the Hospital Universitario Marqués de Valdecilla in Santander, Spain (4). The strain was routinely cultured on blood agar (BA) plates, and Luria-Bertani (LB) broth at 37°C, and frozen at −80°C with 20% glycerol. This strain produces quorum-sensing signals and forms biofilms and is highly cytotoxic in human macrophages and human epithelial cells. The total genomic sample of *S. liquefaciens* isolate HUMV-21 was extracted and purified using the GeneJET genomic DNA isolation kit (Thermo Scientific). The genomic DNA was submitted to Macrogen (Macrogen, South Korea) for PacBio single-molecule real-time (SMRT) sequencing. A single library was prepared for *S. liquefaciens* HUMV-21 and run on one SMRT cell. With a genome size of approximately 5.3 Mb, PacBio SMRT sequencing provided approximately 100 coverage of the entire *S. liquefaciens* HUMV-21 genome. SMRT sequencing initially resulted in 116,360 raw reads, with a mean subread length of 8,671 bp, totaling 1,009,017,802 nucleotides. The generated reads were then introduced into the Hierarchical Genome Assembly Process version 3 (HGAP3), which includes assembly with the Celera Assembler and assembly polishing with Quiver (6). The final complete genome resulted in a single chromosome of 5,326,657 bp, with a total G+C content of 55.2%. A total of 5,844 protein-coding sequences were predicted, of which 22 encode rRNA and 88 encode tRNA. The RAST server (7) predicted coding sequences belonging to 588 subsystems, including 704 involved in carbohydrate catabolism, 287 in protein metabolism, 539 in the synthesis of amino acids and derivatives, 189 in cell wall and capsule synthesis, 254 in RNA metabolism, and 112 in DNA metabolism, including 288 in cofactors, vitamins, prosthetic groups, or pigments, 148 in nucleoside and nucleotide synthesis, 155 in fatty acid and lipid synthesis, 125 involved in virulence, disease, and defense, 178 in membrane transport, 182 in stress response, 73 in phosphorus metabolism, 162 in regulation and cell signaling, 8 in secondary metabolism, 36 phages, and 85 in motility and chemotaxis.

### Nucleotide sequence accession number.

The complete genome sequence of *S. liquefaciens* strain HUMV-21 has been deposited at DDBJ/EMBL/GenBank under the accession no. CP011303.
